# Resilience Design of Healthcare Resources Supply Network Based on Self-Organized Criticality

**DOI:** 10.3390/healthcare8030245

**Published:** 2020-07-30

**Authors:** Liang Geng, Renbin Xiao, Jie Chen

**Affiliations:** 1 School of Science, Hubei University of Technology, Wuhan 430068, China; gengliang@hbut.edu.cn (L.G.); didachenjie@163.com (J.C.); 2 School of Artificial Intelligence and Automation, Huazhong University of Science and Technology, Wuhan 430074, China

**Keywords:** supply network design, resilience, self-organized criticality, avalanche

## Abstract

The healthcare resources supply network design for resilience is an effective way to deal with uncertainty disruption. In this article we propose a model of supply network self-organization evolution, and establish self-organized criticality as a cause of cascade failure. Our main purpose is to keep the system in a resilient range, i.e., critical state. A network structural design with smaller degree distribution exponent can achieve better absorptive capacity at macro level. An interactive rule design with extremal optimization has better adaptive capacity at micro level. Using macro statistic and indicator micro performance indicator, we demonstrate that our design can slow the development to a supercritical state and can improve the resilience of the supply network.

## 1. Introduction

A novel coronavirus (2019-nCoV)-infected pneumonia has been detected in over 10 million patients and has claimed more than half a million lives as of 30 June 2020. As this virus propagates rapidly, any failure in dealing with its prevalence increases the number of infected people. One of our critical tasks is to address the shortage of healthcare resources. In the epidemic situation, desperate need for medical supplies will emerge in a few days. That is why rapid distribution of medical supplies plays an important role in public health management [1]. Due to the lack of healthcare resources, the reliable design of healthcare resources supply network (HRSN) has attracted significant attention [2].

With the development of the epidemic, healthcare resources, such as testing kits, masks, and gloves could be relatively inadequate. If hospitals are faced with a shortage of healthcare resources, an increase in the number of infected people could cause an incredible disaster. Therefore, it is important to optimize the use of the available healthcare resources. Recently, the healthcare resources supply network in most countries is a primary endeavor to maximize the populations’ health and minimize healthcare costs. But considering the uncertainties, it is of greatly difficult to improve the design and the management of healthcare resources supply networks.

HRSN can be defined as a set of interconnected, autonomous agents with self-interested goals that interact to enable the flow of tasks, physical goods, and information [3]. HRSN is becoming more complex and simultaneously more vulnerable to disruptions. The disruption risks are different from the operational risks. The latter are related to inherent uncertainties, e.g., uncertainty in demand, supply and lead times. Despite of a slim chances to occur, the unexpected disruptions, such as epidemic, earthquakes, fires, and even terrorist attacks, could cause a significant business impact [4]. Designing a resilient supply network is an effective way to cope with disruptions [5]. A resilient supply network (SN) has the ability to react to an unexpected disturbance and to return quickly to an original or improved state after a disturbance [6]. Several design strategies have been proposed and studied to optimize supply network performance [7].

In general, supply network design includes the numbers, locations, and capacities of enterprises and the quantity of flow between them. These designs mainly pay attention to improve the network performance, rather than to improve the SN resilience. Disruptions are constituted by internal cause and external cause. The internal cause is the vulnerability of SN, which are influenced by the structure and characteristic of itself. The external cause is emergency, which are difficult to expect. Therefore, the design of resilient SN by identifying the structure and characteristic is a universal method.

A disruption may initially disable one or a few nodes. However, impacts may propagate further through logistics, information flow, and capital flow. Therefore, the failure of any node may cause a catastrophic failure of the whole supply network. As a typical case, the global supply chain of personal protective goods has been disrupted in April 2020.

Self-Organized Criticality (SOC) describes a property that consists of a critical state formed by self-organization at the border of order and chaos. One of the characteristics of SOC is that small disturbances can lead to small or large avalanches, which show a power-law proportion in the size [8]. SOC has become a theory to explain the cascading failure, and can effectively influence the design of supply network to maximize its resilience.

The study is that how to mitigate epidemic impact on the healthcare supply network to control the outbreak of an epidemic. The rest of the article is organized in the following ways. In Section 2, the resilience and the design of HRSN are summarized. In Section 3, the relationship between the resilience and SOC is discussed to elucidate that the resilience range is the self-organizing critical state. In Section 4, a self-organization evolution model is introduced to reveal the HRSN cascade failure mechanism. The principle and the methods of HRSN design, and cases are illustrated in Section 5 and Section 6, respectively. Conclusions are drawn in Section 7.

## 2. Literature Review

### 2.1. Supply Network Resilience

Resilience is regarded as a property that allows SN to react to internal/external vulnerabilities [9], and to guaranty high performance by quickly recovering to an equilibrium state. Tukamuhabwa et al. [10] define SN resilience as “The adaptive capability of a SN to prepare for and respond to disruptions, to make a timely and cost effective recovery, and therefore progress to a post-disruption state of operations—Ideally, a better state than prior to the disruption”. They demonstrated that SN resilience has many characteristics of a complex adaptive system, including adaptation, self-organization, non-linearity, and emergence.

In terms of how SN affecting the resilience, Soni et al. [11] proposed a model using graph theory to measure resilience. In this interpretive structural modeling approach, all the major factors of resilience were taken into account. Depending on the levels of affecting the resilience, major factors are arranged as follows: Collaboration, agility, visibility, risk management culture, adaptive capability, risk and revenue sharing, trust among players, information sharing, sustainability, corporate social responsibility, information security, supply network structure, strategic risk planning, and knowledge sharing.

Despite of some overlapping in definition, the resilience has the following components:

(1) Three core abilities: Absorptive capacity, adaptive capacity, and restorative capacity.

Absorptive capacity plays a role as a buffer in emergencies by resisting the disruption impacts to any further. Adaptive capacity implies the ability of SN to develop different responses to match different threats. This implies that the enterprise can provide an appropriate response to a disruptive event rather than pre-existing response. Restorative capacity is the ability of recovering to its original performance level, which greatly depends on the resilient measures, such as adding or changing relations between supply and demand and introducing new firms.

(2) A supporting condition: Vulnerability.

Vulnerability is another factor which concerns us. Serious disturbance can make the operation of SN deviate from the normal state.

Flexibility reflects the adaptive capability. Flexible strategy is a kind of “alteration according to changes” strategy. Robustness reflects the absorptive capability. It characterizes the stability of the system. Robust strategy is a kind of “responding to changes with invariability” rigid strategy. Robustness is not adaptive, while resilience is adaptive. This adaptability refers to the ability to quickly return to the initial state or a more favorable state after an interruption. Robustness is impact resistant. Resilience is the ability to recover quickly from impact.

Flexible strategy and robust strategy have their own advantages and limitations. Facing the complex and changeable uncertain environment, it should have both adaptability and stability. “Temper force with mercy” or “Couple hardness with softness” can effectively avoid risks. From this comes the concept of resilience. Resilience includes flexibility and robustness, and has restorative capability [12,13].

**Figure 1:**
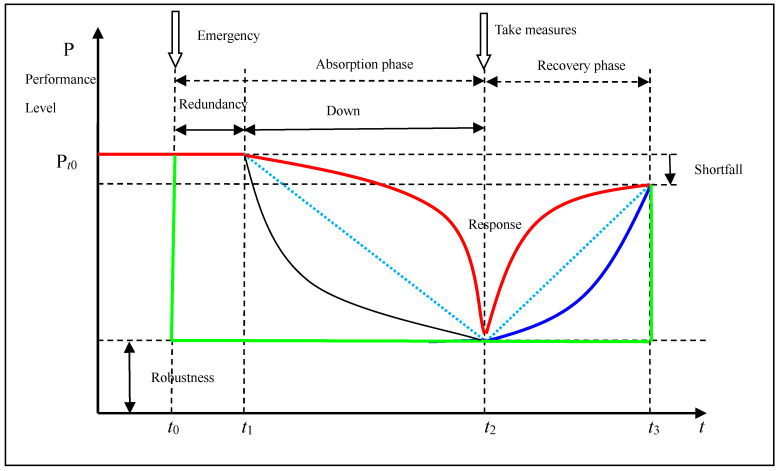
Measure of supply networks resilience.

How to measure the resilience of SN is a hot topic. For instance, Munoz and Dunbar [14] built a multi-dimensional and multi-level SN resilience index. The resilience triangle is the most accepted method to assess the resilience. Resilience is depicted as a process in which a SN experiences disruptive events and makes a series of responses. As shown in Figure 1, *t*_0_ is the time when a disruption begins, *t*_0_*–t*_1_ is the buffer phase, *t*_0_*–t*_2_ is the absorption phase, *t*_2_*–t*_3_ is the recovery phase.

It is using the degree of curvature of performance curve to measure the resilience of SN. As shown in Figure 1, convex curve has better resilience than the concave curve. So, we propose a indicator to measure the resilience from microscopic performance change.

(1) $$R(t)=1-\frac{\int_{t_{0}}^{t_{3}}\left[P(t_{0})-P(t)\right]dt}{P(t_{0})\times (t_{3}-t_{0})}$$

Therein, $\int_{t_{0}}^{t_{3}}\left[P(t_{0})-P(t)\right]dt$ denote the performance decline which is the area at the above of the performance curve. This method can evaluate not only the loss in performance after a disruption but also the time it takes to recover.

In addition, we can measure the resilience from the macro statistics. (2) express that the smaller probability of large-scale avalanche incidents, the bigger resilience of SN. Therein, $\tilde{s}$ denote a big avalanche size, $P(\tilde{s})$ denote the probability of this avalanche size.

(2) $$P_{1}(\tilde{s})<P_{2}(\tilde{s}),\;\mathrm{so}\;\mathrm{that}\;R_{1}>R_{2}$$

### 2.2. SN Resilience Design

The interests in SN design are driven by the economic benefits. The design of Altiparmak et al. [15] is to satisfy the customer demand with minimum cost, which presents a steady-state genetic algorithm for the design of a single-source, multi-product, multi-stage SN. The design of Pishvaee et al. [16] proposes a mixed-integer linear programming model for handling the inherent uncertainty of input data in a closed-loop SN. The design of Wang et al. [17] is to captures the trade-off between the total cost and the environment influence, which presents a multi-objective optimization model for the classical facility location problem. The design of Nickel et al. [18] is to maximize the total economic benefit and minimize the overall cost, which presents multi-stage stochastic mixed-integer linear programming method. The design of Carvalho et al. [19] is to improve the SN resilience by evaluating different scenarios, which comprehend the mitigation strategies through the simulation analysis of Portuguese automotive SN. These designs lack in SN resilience although which can optimize from different aspects. The resilient SN design should consider not only the benefits but also the risk.

Some scholars studied how to improve the resilience, although there has not been any consensus. As Sheffi [20] considers that building redundancy is one of the most direct methods for creating resilience. On the contrary, the study by Kim et al. [21] shows that increasing redundancy may not improve resilience by adding extra nodes or arc. Redundancy is one of the key points in creating robustness. So, redundancy can create resilience.

**Figure 2:**
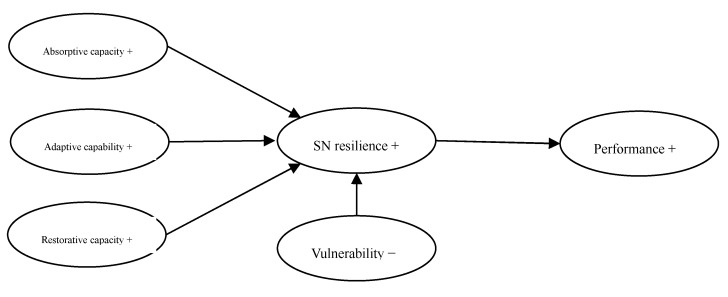
Supply chain design strategies for supply network (SN) resilience.

Pettit et al. [22] argue that SN resilience increases as capabilities increase and vulnerabilities decrease. As shown in Figure 2, SN can be designed from two aspects to improve resilience. On one hand, SN reduces vulnerability. On the other hand, SN enhances the absorptive capacity, adaptive capacity, and restorative capacity. As to spreading the negative impact of the emergency, it depends on the macro SN topology structure and the microscopic rules of interaction between enterprises.

Network topology optimization is to search the appropriate network structure, which can reduce the effects of cascading failure. Rivkin and Siggelkow [23] summarize some basic network structure. Based on this concept, Kim, et al. [21] Supply network structure can be divided into 4 classes:

(1) Block-diagonal.

This SN structure has clusters of nodes between the source and sink, where connections occur within clusters but not between clusters. It comprises a final assembler and various module suppliers, each of which is fully responsible for designing and manufacturing the assigned module, such as personal computer SN [24].

(2) Diagonal.

In this SN structure, most of the nodes in between the source and sink can be partitioned into subsets, in which the connections primarily occur across different tiers, such as military logistics networks [25].

(3) Centralized.

In this structure, a few nodes connect to (almost) all other nodes, while the other nodes link only to a few highly central nodes. In this “winner-take-all” structure, the top-tier suppliers plan and manage all the necessary steps. Such as textile supply network in the Prato (Italy) [26].

(4) Scale-free.

In this structure, the node degree distribution follows a power-law. A few nodes contain disproportionately too many connections, while most of the other nodes have only a few connections. That is a small number of “core” firms jointly control and manage larger numbers of “peripheral” firms, such as Toyotacity [27] and the aerospace industry around the Seattle region [28]. Comparing four kinds of structure, the scale-free network has better robustness, so it is more resilience in one way.

In 1996, USA branch of Honda launched a set of supply chain solutions named “MOVE”, which can change the order according to the actual need. It reduces the time of order and inventory, and has saved a lot of money. It shows the importance of the interactive process. Interactive rules optimization is to adjust the relationship between satisfaction and saturation, which can slow down the development to supercritical.

## 3. The Relationship between Self-Organized Criticality and Resilience

The concept of self-organized criticality was proposed by Bak when he studied system complexity. From the point of view of the function mechanism, interaction is the source of system evolution. SOC explain the behavior characteristic of the complex system which contains a large number of short range interaction components. According to this view, a system can be divided into the subcritical state, critical state, and supercritical state. Under normal condition, systems naturally evolve towards the critical state. However, systems may enter the supercritical with a massive avalanche facing to disruption. The supply chain resilience is described as a macroscopic property that generates from self-organizing behavior of each enterprise on the microlevel [29]. Korosh proposes a social model of spontaneous self-organization generating criticality and resilience [30].

We consider that resilient range is self-organizing critical range based on the following:

(1) In the self-organized critical state, system has the highest efficiency, which can both benefit and risk.

SN has been considered as a complex adaptive system. Both SOC and complex adaptive system theory regard complexity originated from the “edge of chaos”. The difference lies in the methodology. SOC is a new kind of statistical theory. Complex adaptive system theory is trying to find mechanism of evolution from micro to macro outside statistical theory. In self-organizing critical state, system has enough stability to maintain its own survival, also have enough energy for development. In this condition, system not only has better adaptive capacity but also can effectively reduce vulnerability.

(2) In the self-organized critical state, the avalanche sizes can reflect the vulnerability.

The essence of self-organized criticality is the global response of space and time caused by small fluctuation. Vulnerability is to look at resilience from an opposite viewpoint. The essence of the vulnerability is the cascading failures phenomenon. This is similar to the collapse of the chain reaction when the system is in SOC. Based on the facts that:

- Vulnerability and SOC have the characteristic of large-scale collapse.
- The sizes and frequency of collapse is power-law distribution.
- Each component has a strong correlation.
- Exciting cause is beyond the threshold.

So, in the self-organized critical state, different avalanche size can reflect resilience from the opposite.

(3) The performance of system fluctuates in the self-organized critical state.

The evolution process of SN can be explained as follows. On one hand, in order to improve efficiency, the vulnerability increases and the large-scale avalanche more easily occurs. On the other hand, small avalanches release some of the load, that relieve the development to supercritical. It will absorb some influence of disruptions and reduce the risk of accidents. The two opposite forces make the performance of a system fluctuate in a certain range. After a small-scale avalanche, the performance will descend, but then the performance will slowly ascend due to the ability of self-organization.

**Figure 3:**
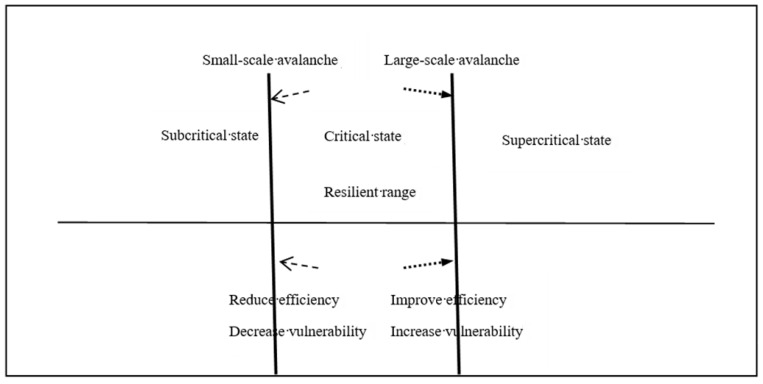
The relationship between Self-Organized Criticality (SOC) and resilience.

Resilient supply network is neither in a stable subcritical state, also is not in the chaotic supercritical state, rather in the self-organized criticality state. It is the state that not only has local interest maximization, but also prevents whole the system from collapsing. As shown in Figure 3, the resilient range is the self-organizing critical range.

The resilience of supply network based on SOC is expressed as follows. In order to maximize their own interests, the enterprises will maximize actual operation ability, that is a full load operation.

**Figure 4:**
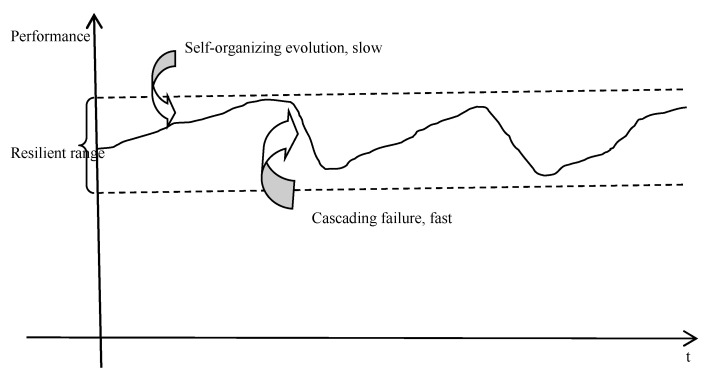
The resilience of supply network (SN) based on SOC.

The supply network will develop from the subcritical state to the critical state. If we seek efficiency blindly, vulnerability will increase. The supply network will develop to the supercritical state. As shown in Figure 4, the load grow process is slow and dynamic. When the supply network reaches a certain load, cascading failure may occur. Cascading failure is a fast dynamic process. Slow self-organization process and cascading failure process alternating fluctuates in a certain range, which constitute the resilience of supply network to balance the benefits and risks. In the resilient range, the supply network not only has strong absorptive capacity, adaptive capacity, and restorative capacity, but also can effectively reduce vulnerability.

In real systems self-organization as an emerging property can rarely be fully predicted. However, local rules can be designed to adjust the behavior of self-organization by taking advantage of its characteristics. The design of local rules is to control the performance within the resilient range as Figure 4. Overall resilience emerges through the regulation of local rules. Resilience is the tendency to change to remain within a stability domain, continually changing and adapting, yet remaining within critical thresholds such as the resilient range as Figure 4. As the system approaches thresholds, it has to be controlled.

## 4. Self-Organized Criticality of Supply Network

### 4.1. The Model of Supply Network Self-Organizing Evolution

The purpose of the supply network operation is to profit and to meet customer requirements, and ultimately to achieve good performance. In the supply network, each enterprise abstract as a node, and the relationship among supply interactions such as integrated logistics consisting of information flow, cash flow, material flow, etc., abstract as side.

**Figure 5:**
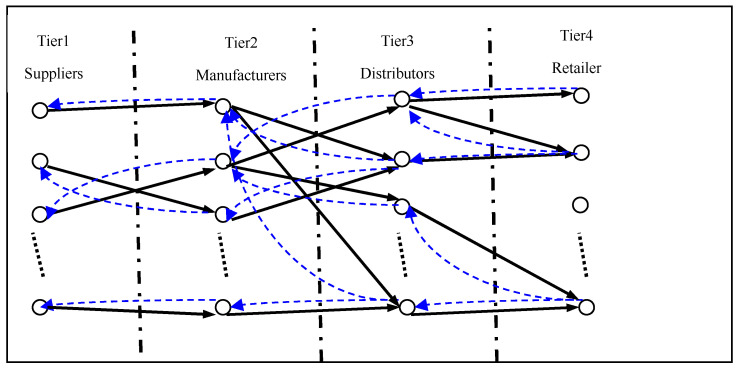
Structure of a supply networks.

From a logistics perspective, supply network is a hierarchical network. The high-level customer sends information of purchasing expectation to the low-level supplier, and low-level supplier sends goods according to the actual purchasing quantity to the corresponding high-level customer. Expected purchasing quantity and actual purchasing quantity are integrated logistics generated during interaction between supplier and customer [31], as shown in Figure 5. The names of all the variables in the model are defined as shown in Table 1.

Definitions of the variable in the model as shown in Table 1.

#### 4.1.1. The Parameters of the Model

Each enterprise has certain practical operation ability at every moment, known as load. Set as *h_i_ =* *αk_i_*^1−*η*^, 0 < *η* < 1, *k_i_* is node degree value, *α*, *η* are variables. Each enterprise also has a maximal ability of load, called capacity. Set as *z_i_* = (1 + *θ*) *h_i_*, 0 < *θ* < 1. For simplification, when *t* = 1, let expected purchasing quantity equal to load amount of customer. Σ*e_ij_*(*t*) = *h_i_*(*t*), the expected purchasing quantity from *i* to *j*, *e_ij_*(*t*) = *h_i_ × h_j_/*Σ*h_j_*.

With changes in the actual operational capabilities, customers want to buy the amount to match their actual operating capabilities to produce. However, the actual purchasing quantity is limited by the supplier’s load, and can only be distributed in the range of less than or equal to the load to meet the needs of customers. The distribution principle of the actual purchasing quantity is a crucial problem in the whole process of interaction.

If the actual purchasing quantity of the superior customer is not more than the supply ability of the supplier, the customer’s purchasing quantity is assigned according to the customer’s expectation, *r_ij_*(*t*) = *e_ij_*(*t*). If not, the customer’s purchasing quantity is assigned according to the supply ability of the supplier. Distribution probability is:

(3) $$p_{i}(t)=\frac{E_{i}(t)}{\sum_{i}E_{i}(t)}$$

For each pair of supply (side), its satisfaction is the ratio of the actual purchasing quantity and the expected purchasing quantity:

*r _ij_*(*t*) = *e_ij_*(*t*)/*s_ij_*(*t*) (4)

For a node, its satisfaction can be expressed as:

*R_i_*(*t*) = *S_i_*(*t*)/*E_i_*(*t*) (5)

#### 4.1.2. Normal Self-Organization Evolution

Enterprises will adjust their expected purchasing quantity and actual purchasing quantity to improve their satisfaction by themselves. The load of customer is the sum of expected purchasing quantity, the load of supplier is the sum of actual purchasing quantity.

**Figure 6:**
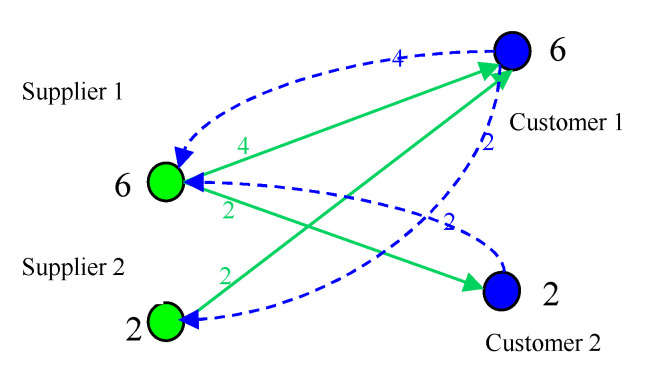
Initial situation.

**Figure 7:**
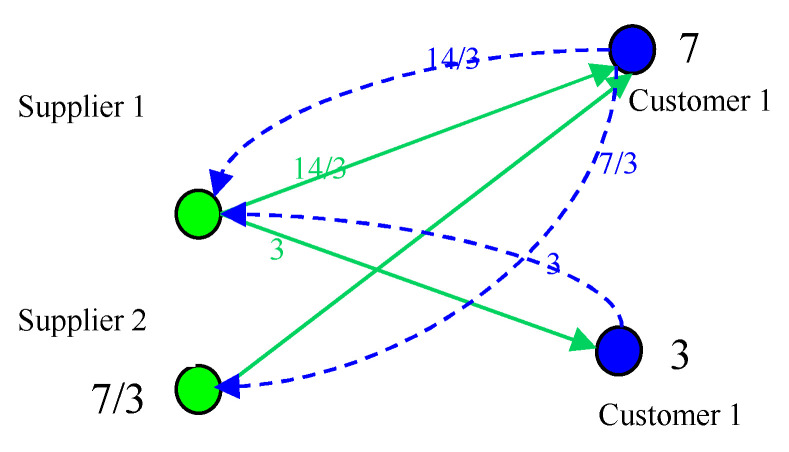
The case of self-organizing evolution.

Assuming that the capacity of supplier 1 and supplier 2 respectively is *z*_S1_ = 8, *z*_S2_ = 4, the capacity of customer 1 and customer 2 respectively is *z*_c1_ = 8, *z*_c2_ = 4. Initially, the expected purchasing quantity of customer 1 and customer 2 respectively is 6 and 2. As shown in Figure 6, the load of supplier 1 and supplier 2 is 6 and 2, both satisfaction are 1. At this time due to customer satisfaction to achieve the desired effect, customer will increase the expected purchasing quantity. Both customer 1 and customer 2 expected purchasing volume increased by 1, respectively, 7 and 3. As shown in Figure 7, the load of supplier 1 and supplier 2 is 23/3 and 7/3. The load of each enterprise has increased, more and more close to the capacity. That is to say, with the enterprise self-organization to improve satisfaction, the system is becoming more and more critical. This is a slow dynamic process.

The self-organization evolution of the system is as follows:

Assuming *t* moment, the satisfaction of customer *i* is *R_i_*(*t*), if it is greater than a threshold value of *S*th. Then the *t +* 1 moment, it is the expected purchasing quantity:

*E_i_*(*t*) = min{(*E_i_*(*t*) + *ω*) *× h_i_*, *z_i_*} (6)

The *ω* is a constant, adjust the specific satisfaction of the time, the increase in expected purchasing quantity.

According to (3) distribution, the actual purchasing quantity is obtained with the change of the expected purchasing quantity. With the increase of system satisfaction, network load also gradually increases, more and more close to the capacity. Systems are becoming more and more close to the supercritical state, which can easily cause a large avalanche.

#### 4.1.3. Cascade Failure

The supply network works well only when enterprises’ load capability is lower than capacity. Otherwise the company suspended it in a supply network function to wait for recovery. The enterprises will distribute some of its load to related enterprises. This will increase the load of other enterprises, and may cause a cascading failure, resulting in a large-scale supply network crash.

Based on the self-organization evolution, the cascading failure process of the system is as follows:

1. Occurrence of unexpected events: The moment *t* a node *i* failure, the load of the node reached or exceeded the capacity of *z_i_*, the node crashes, bear the part of the function assigned to the interrelated enterprise to complete.
2. Load distribution: When the node failure, the node will be assigned to the average distribution of connected nodes, the node in the network disconnect to other nodes.
3. After the load is pushed and the node *j* has more than its own capacity, the node *j* fails.
4. Repeat 2–3 until there is no node failure, the system reaches a stable state.

The basic operation mechanism of this model is the interaction between the enterprises. Enterprises self-organization adjust their supply relations to change their expected purchasing quantity, the change of expected purchasing quantity alters the actual purchasing quantity, and enhance the satisfaction. The incremental change increases enterprise load. The system becomes more and more critical, which leads to the occurrence of cascading failure.

### 4.2. The SOC of Cascade Failure

**Figure 8:**
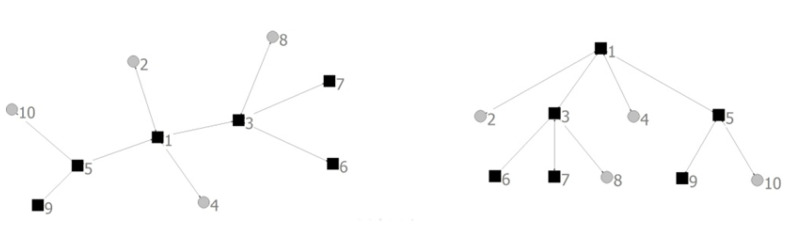
Branching process.

Branching process [32] method is a useful tool to study the avalanche dynamics, which is valid when avalanche trails do not form any loop. Each avalanche can map a corresponding tree. The node where an avalanche is triggered is viewed as the originator of the tree. The other nodes in the avalanche correspond the descendants. The avalanche proceeds can be identified with that of trees grown. The avalanche size *s* is the tree size. As shown in the Figure 8, *s* = 6.

The threshold of node enterprise determined by degree of node: *z_i_ = k_i_^1-η^*, (0 < *η* < 1). We consider the degree distribution of SN follow *p_d_*(*k*)*~k ^−γ^*. The branching probability *q*(*k*) that a node topple to its adjacent *k* nodes is the only parameter of a given branching process, which is composed of two factors:

(1) *q*_1_(*k*) is the probability that a node has the threshold *k*−1 < *z_i_* < *k.* Only when the height of a node is *k*−1, load transfer can be triggered.

(7) $$q_{1}(k)=\sum_{k^{\prime }=\left\lceil {\left(k-1\right)}^{1/(1-\eta )}\right\rceil }^{\left\lfloor k^{1/(1-\eta )}\right\rfloor }kp_{d}(k)/<k>∼k^{{}^{\left(1-\gamma +\eta )/(1-\eta \right)}}$$

(2) *q*_2_(*k*) is the probability that the node has height *k*−1 when gaining the load from one of its neighbors. Because of every number of load from 0 to *k*−1 is equally probable.

*q*_2_(*k*) = 1/*k* (8)

The branching probability *q*(*k*) for large k is given asymptotically as:

(9) $$q(k)=q_{1}(k)q_{2}(k)∼k^{-\gamma^{\prime }}\;[\gamma^{\prime }=(\gamma -2\eta )/(1-\eta )]$$

Using the independence of the branching from different parent-nodes, one can derive the following relation for distribution of avalanche size *p*(*s*) (i.e., the distribution of tree size).

(10) $$p(s)=\sum_{k=0}^{\infty }q(k)\sum_{s_{1}=0}^{\infty }\sum_{s_{2}=0}^{\infty }\cdots \sum_{s_{k}=0}^{\infty }p(s_{1})p(s_{2})\cdots p(s_{k})$$

By introducing the generating functions of *q*(*k*): $L(w)=\sum_{k=0}^{\infty }q(k)w^{k}$ and the generating functions of *p*(*s*): $P(y)=\sum_{s=1}^{\infty }p(s)y^{s}$, this relation can be written in a compact form:

(11) P(y)=yL(P(y))

Then *w* = *p*(*y*) is obtained by inverting *y* = *p*^−1^ (*w*) = *w*/*L*(*w*).

The avalanche size *s* can be expressed by generating functions of *p*(*s*). Mean value avalanche size can get from generating functions of *p*(*s*):

(12) $$<s>=\sum_{s=1}^{\infty }p(s)s=P^{\prime }(1)$$

From (5), $P^{\prime }(y)=\frac{L(P(y))}{1-yL^{\prime }(P(y))}$, combine (6) thus:

(13) $$<s>=P^{\prime }(1)=\frac{L(P(1))}{1-L^{\prime }(P(1))}=\frac{L(1)}{1-L^{\prime }(1)}$$

When *L’*(1) = 1, <*s*> is diverging, which means happening large-scale avalanche.

(14) $$C=L^{\prime }(1)=\sum_{s=1}^{\infty }kq(k)=1$$

*C* = 1 is the critical point which can cause large-scale cascading failure [33]. It means that the model of SN reveals the basic principle of cascading failure and shows the SOC.

## 5. Design for SN Resilience

Cascading failure represents the vulnerability of SN and the satisfaction of system represents the performance of SN. Our design is to make the vulnerability smallest and the satisfaction largest. It is equivalent to make it reach the critical state where local profit is largest, but it has no systematical collapse entirely.

### 5.1. Network Structure Design

The network’s own absorptive capacity is determined by the network structure. So it affects the scale of avalanche which is the size of the vulnerability. The motivation of network structure design is to find the appropriate network structure which could slow down the speed of the system changing to supercritical state during the loading distribution.

Since:

(15) $$\sum_{s=0}^{\infty }a_{s}y^{s}∼{\left(1-y\right)}^{\phi }\;;\;a_{s}=\frac{\Gamma (s-\phi )}{\Gamma (s+1)\Gamma (-\phi )}∼s^{-\phi -1}\;(s\to 0)$$

When *P*(*y*) ~ (1−*y*)*^Φ^*, *p*(s) ~ s^−*Φ*−1^.

From $y=P^{-1}(w)=w/L(w)$, set $\frac{\partial P^{-1}(w)}{\partial w}=0$, generating function *L*(*w*) is singular at *w =* 1. the expansion of *L*(*w*) around *w =* 1 is given as:

(16) $$L(w)≃1-(1-w)+\{\begin{array}{cc} {\left(1-w\right)}^{\gamma^{\prime }-1} & 2<\gamma <\gamma_{c}\\ -{\left(1-w\right)}^{2\ln(1-w)} & \gamma =\gamma_{c}\\ {\left(1-w\right)}^{2} & \gamma >\gamma_{c} \end{array}$$

where:

*γ*’ = (*γ −* 2*η*)*/*(1 − *η*), and *γ_c_* = 3 *− η.* (17)

From the relation between *L*(*w*) and *P*(*y*), we obtain the distribution of avalanche size *p*(*s*)~*s^−τ^* [34]

(18) $$p(s)∼\{\begin{array}{cc} s^{-(\gamma -2\eta )/(\gamma -1-\eta )} & 2<\gamma <\gamma_{c}\\ s^{-3/2}{\left(\ln s\right)}^{-1/2} & \gamma =\gamma_{c}\\ s^{-3/2} & \gamma >\gamma_{c} \end{array}$$

The Formula (16) shows avalanche size distribution obeys power-law distribution, which means the times of happening of small scale of avalanche are in a majority, and the times of large scale are in a minority respectively. It is a common phenomenon for supply network in the real case.

**Figure 9:**
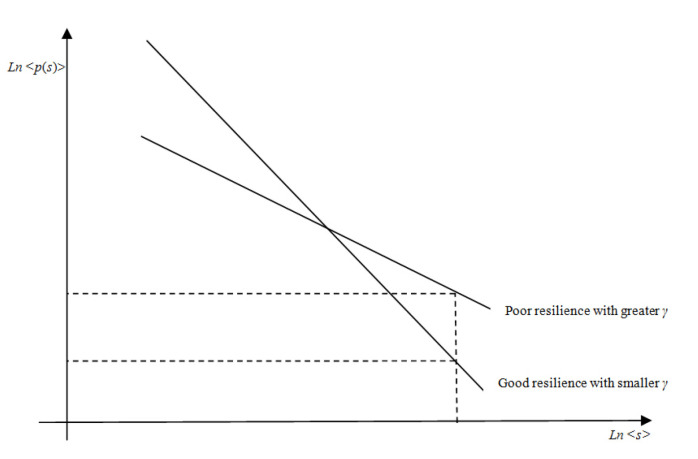
The resilience based on avalanche size distribution.

The avalanche size distribution is a measure for the resilience. The small probability of large scale avalanche shows the supply network possesses a relatively strong absorptive capacity to face the interruption, that is to say it has stronger resilience. The index of the node degree distribution of supply network has a critical number *γ_c_*. When 2 < *γ* < *γ_c_*, the index *τ* increases as *γ* decreases. As shown in Figure 9, the probability of large scale collapse decreases when *γ* decreases. It shows that fault influence is reduced and the resilience is better. Because the major loading is supported by the hub node, the SN has some resistance for the fault. When *γ* > *γ_c_*, the index *τ* = 1.5, which means the distribution of the failure scale is independent of the connecting of node. The scale of avalanche is not controlled by the index of SN node degree distribution, system in the supercritical state.

As shown in Figure 9, the supply network resilience was better with smaller γ and larger τ. 

### 5.2. Design for Interaction Rules

Every enterprise is seeking itself profit maximization interacting with others. This simplex self-optimization will usually increase the spread of Cascading failure. The motivation of the design of interaction rules is to increase customer satisfaction and make the system away from the supercritical state avoiding the large scale avalanche.

**Figure 10:**
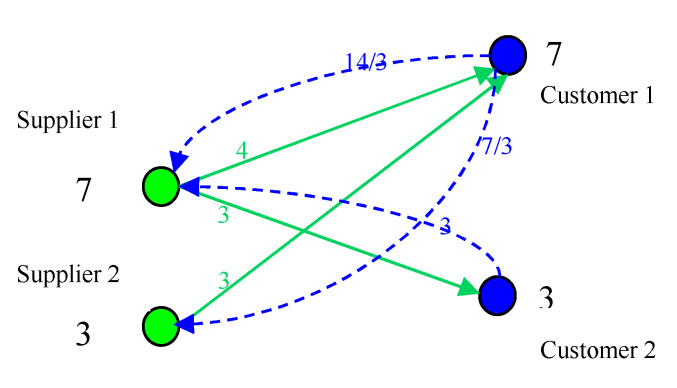
The case of self-organizing optimization.

Saturation is systematically considered during the interaction process. *A*_1_ = 0.958, *A*_2_ = 0.58 are the original Saturation of suppliers. After changing the interactive rule, as shown in Figure 10, suppliers’ saturation are *A*_1_ = 0.875, *A*_2_ = 0.75. In the condition of maintaining customer satisfaction, saturation of the supplier1 decreases drastically. Saturation of the supplier 2 is still away from the critical state although it increases a bit. It shows that the proper optimization of interactive rule can release cascading failure.

We will design the interactive rule base on SOC. The criticality in SOC is different from the stationary statistical mechanics, in which the critical point is the place where the behavior of system or the structure changes drastically. Such as water from liquid state to solid state, the parameter is the temperature. The phase transition is obtained from adjusting some parameter in the system. But the factor of drive system to achieve the critical state is internal dynamics mechanism without any parameters adjustment.

Extremal optimization (EO) algorithm is based on SOC theory, without adjusting any parameter. From the connection of internal variables in the problem, regarding the process of optimization as the evolution of complex system, EO make the system always evolve to the optimized structure only from the variation of the worst element. EO is a “deleting worst” algorithm. The evolution mechanism increases the excellent individuals and have rapid convergence and good accuracy.

Introduce the EO algorithm to the interactive rule.

Define the fitness of node *j_u_*:

*fit_ju_ = k_ju_ × R_ju_/A*(*j_u_*) (19)

Objective function:

max(*obj*_0_) = ∑*fit_i_* (20)

(1) For the failure node *i*, compute the fitness of nodes connected with it.
(2) The node *i*_0_ with worst fitness will be optimized by EO. Taking its nearby nodes as its neighborhood, the actual purchase quantity from nearby node *j* will become:
  *s_ji_*_0_ (*t* + 1) = (1 + *fit_i_*_0_) *s_ji_*_0_ (*t*). (21)

(3) If the customer’s satisfaction *R_i_*(*t*) is larger than a threshold *S*th, then its expecting purchase quantity at time *t +* 1 becomes:
  *E_i_*(*t*) = min{(*E_i_*(*t*) + *ω*) *× h_i_*, *z_i_*}. (22)
  Here, *ω* is a constant, adjusting the expecting purchase quantity.
(4) Compute the objective function after optimization. If *obj*_0_(*t* + 1) > *obj*_0_(*t*), then max(*obj*_0_) *= obj_0_*(*t* + 1) or else max(*obj*_0_) *= obj_0_*(*t*).
(5) When cascading failure occurs, node *i* will push the loading to the nearby nodes. Consider three factors: 1 saturation *A_j_*; 2 satisfaction *R_j_*; 3capacity *h_j_*. The distribution rule is as follows:
  *h_ju_* = *h_ju_* + *h_i_* × *fit_ju_*/∑*A(j_u_*). (23)

(6) Repeat (1)–(5) until the cascading failure ends.

## 6. Case Study

### 6.1. Case Background

**Figure 11:**
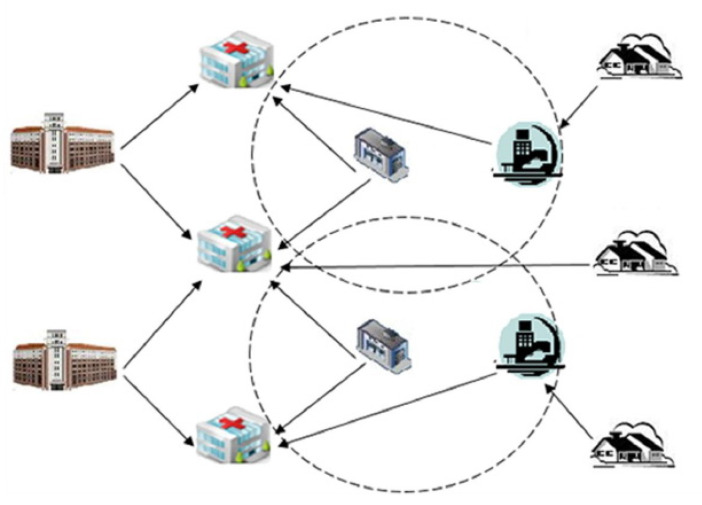
The sample of healthcare resources supply network.

To illustrate the design can effectively improve the resilience, the methods were applied to the SN-1 and SN-2 such as the Healthcare Resources Supply Network in Figure 11 [2]. It involves (1) Backup medical supplies distribution centers, (2) Hospitals, (3) Medical supplies distribution centers, (4) The set of the affected areas. As shown in Figure 11, patients are transferred to the nearest hospital via transfer points or directly. Each hospital can receive medical supplies from several medical supplies distribution centers. The part of hospital needs that is not received from medical supplies distribution centers can be received from backup medical supplies distribution centers [2]. The degree distribution of them obeys power law distribution. The parameters of network structure are shown in Table 2.

### 6.2. SOC in the HRSN

We observe whether there is a critical value in large cascading failure according to Section 4.1.3 method. Using a simple parameters: *α =* 1, *η =* 0, so *h_i_* = *k_i_*, *z_i_ =* (1 + *θ*) *k_i_*. Use the ratio of numbers of failure node and numbers of total nodes to measure the size of the cascading failure *R* = *n*/*N.*

**Figure 12:**
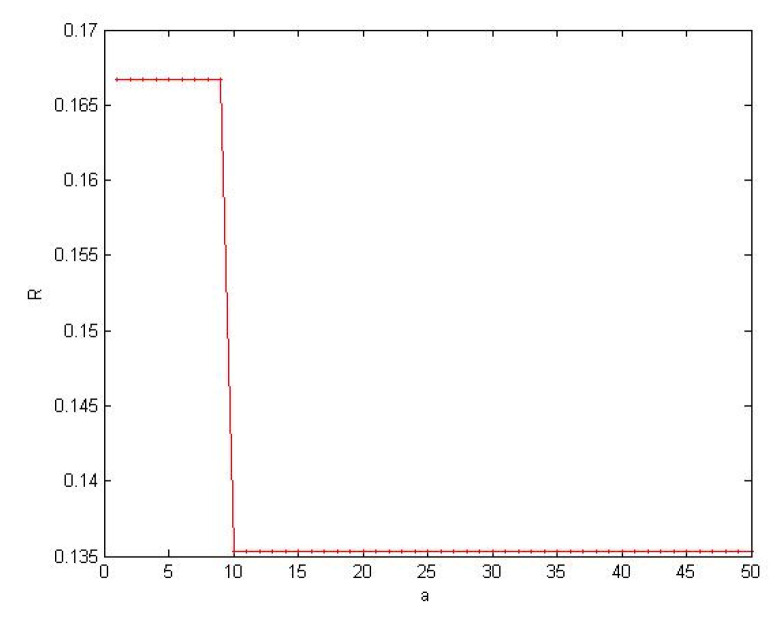
SOC in SN-1.

The results through the simulation as shown in Figure 12, shows that there is a critical value making the network have large-scale cascading failure. The conclusion is same as the Formula (14).

The following experiment verifies the cause of self-organized criticality from the micro level.

**Figure 13:**
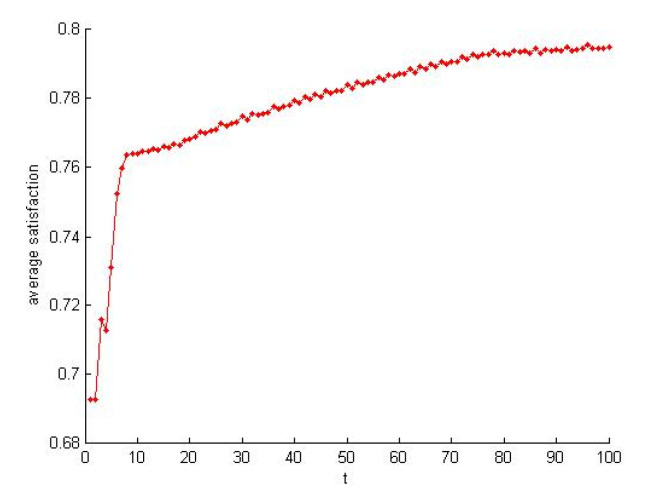
Satisfaction increased in the self-organization evolution.

**Figure 14:**
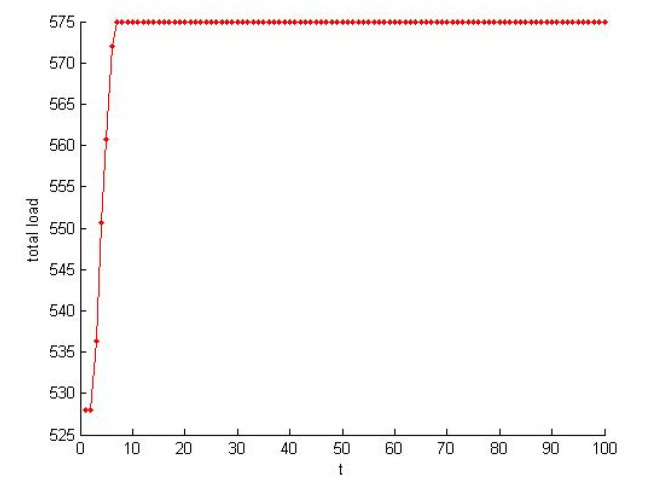
Load increased in the self-organization evolution.

According to the Section 4.1.2, the self-organization evolution of SN-1 is a slow dynamic process in which the satisfaction increases. As shown in Figure 13 and Figure 14, the load also increases rapidly to a certain extent and tends to be steady. The self-organizing behavior makes the supply network develop in the direction of supercritical state. The process of satisfaction enhancement is the process of increasing vulnerability.

**Conclusion** **1.** *The cascading failure of supply network possesses self-organized criticality. The reason of cascading failure is the development of self-organizing evolution toward the supercritical state*.

### 6.3. The Influence of Network Structure Design for Resilience

In order to verify the influence of structure design for resilience, we performed a statistical experiment of the avalanche size. According to the Section 4.1.3, we randomly selected failure node to observe the size of the cascading failure, namely the size of an avalanche. We did the test 1000 times for statistical analysis and made a log–log graph of the avalanche size of probability distribution.

**Figure 15:**
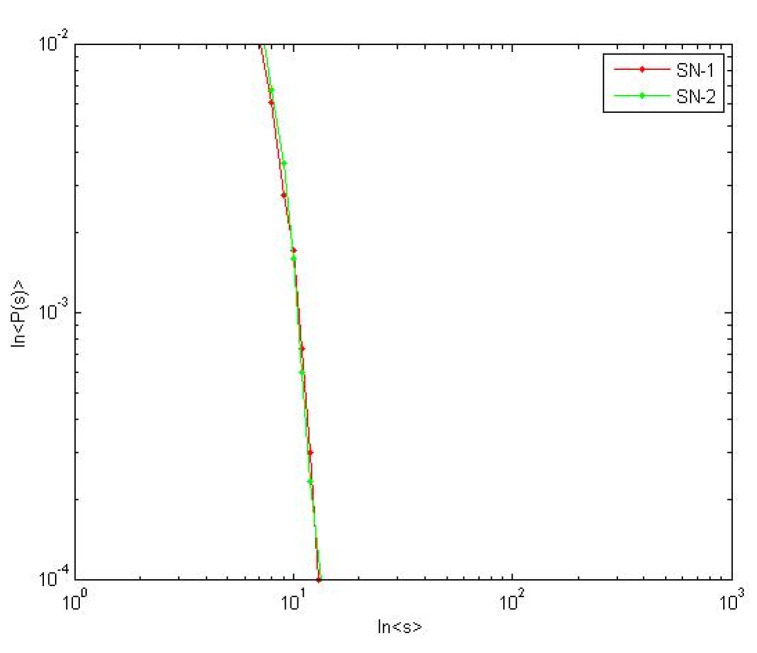
Avalanche size in supercritical state.

Set *η* = 0.75. The avalanche size of two supply networks is appropriately same as shown in Figure 15. Same results are obtained by repeating trials. From the formula (17), *γ_c_ =* 3 − *η*, we can get *γ_c_ =* 2.25. Although *−γ*_1_ and *γ*_2_ are different, they are both greater than *γ_c_*. Thereby the case *γ > γ_c_* in Formula (18) is verified. This result implies that *γ* loses the control for resilience of supply network in supercritical state. At this state, the probability of a massive avalanche is the largest.

**Figure 16:**
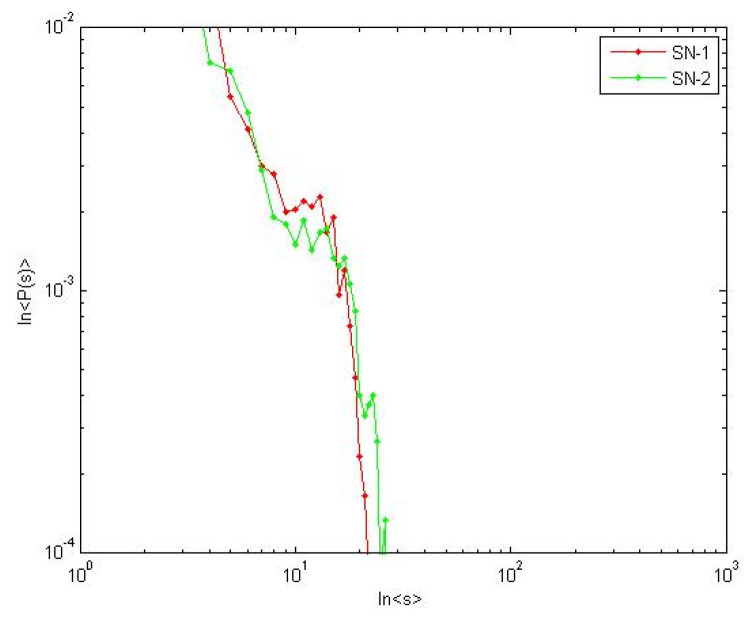
Avalanche size in critical state.

To examine the case *γ* < *γ_c_*, we set *η =* 0.1, the power law index *γ*_1_ and *γ*_2_ are both less than *γ_c_ =* 2.9. As shown in Figure 16, different avalanche sizes obey the power law distribution. Thereby the case *γ* < *γ_c_* in Formula (18) is verified. We used macroscopic statistical indicator in Section 2.1 to measure the resilience.

(24) $$P_{1}(\tilde{s}=30)<P_{2}(\tilde{s}=30)\;so\;R_{1}>R_{2}$$

At this moment, *γ*_1_ *< γ*_2_, *τ*_1_ *> τ*_2_. SN-1 has a smaller probability of a massive avalanche, i.e., better resilience. The result is consistent with analysis in Section 5.1.

**Figure 17:**
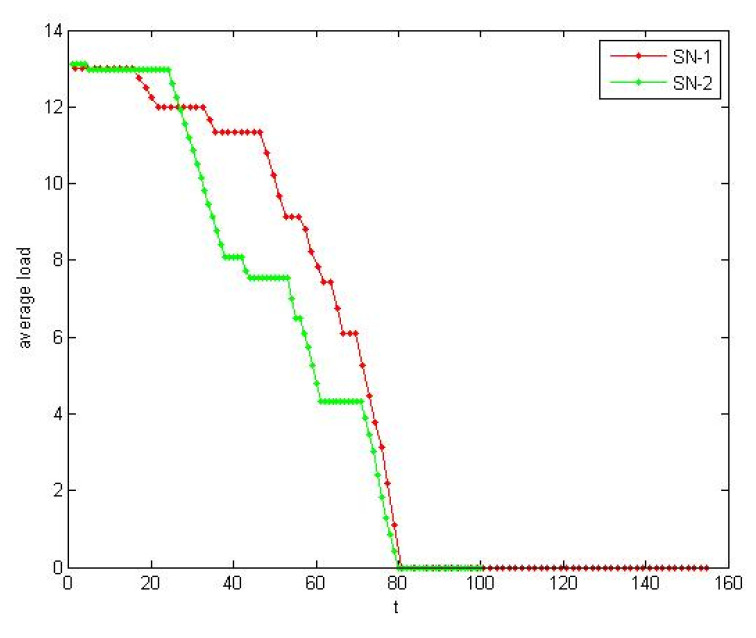
The contrast of absorptive capacity.

**Figure 18:**
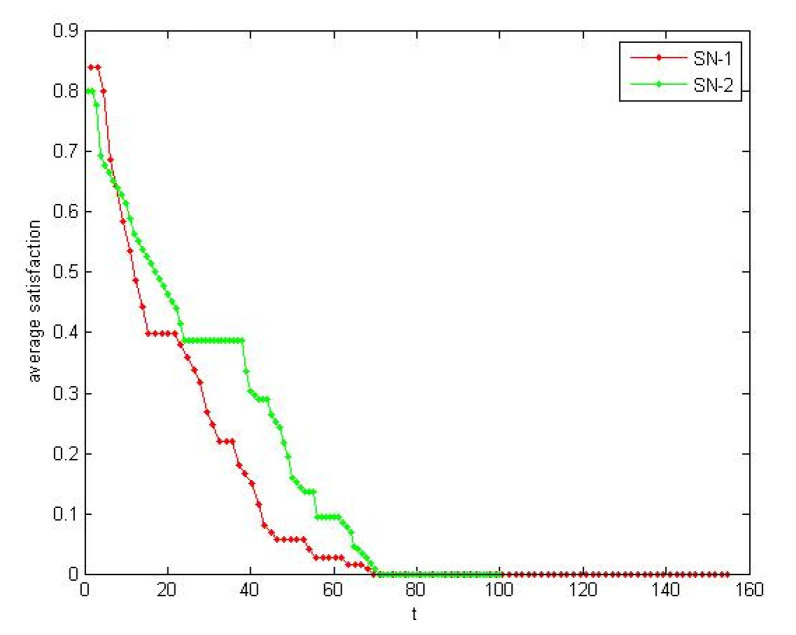
The contrast of adaptive capacity.

Moreover, we observed the influence of performance change to resilience from microscopic. According the method of 4.1.2, we selected lower supplier failure node in the two SNs respectively. *i*(SN1) = 25; *i*(SN2) = 26. Due to the number of nodes being different, we used the average quantity. The change of the average load and the average satisfaction over time of the two SNs are shown in Figure 17 and Figure 18. The rectangle method $\int_{a}^{b}f(x)dx\approx \frac{b-a}{n}(y_{1}+y_{2}+y_{n})$ is used to approximate calculate area above the performance curve. We used micro performance indicator in Section 2.1 to measure the resilience. R_P_(SN1) = 0.732, R_P_(SN2) = 0.621.

Average load implies the overall performance of supply network. More slowly average load of SN-1 decreases, the better absorptive capacity we have. Average satisfaction implies the agility of enterprise to the environment changes. Average satisfaction of SN-1 decrease faster, which illustrates SN-1 has better adaptive capacity.

The reasons of the network structure causing cascading failure: The hub node in redundancy SN has strong operational capacity (load), with more links. When the interrupt occurs, scale-free SN is conducive to disperse load, delaying systems tending to supercritical state.

The resilience of SN-1 is better than SN-2. We verified theoretically Kim’s [21] conclusions: “The more closely a supply network follows a power-law for the degree distribution of the nodes, the more resilient the supply network will become.” and further refine the conclusion.

**Conclusion** **2.** *Ceteris paribus, for non-core node failure, the network has higher heterogeneity with smaller γ, and has the better resilient. The resilient supply network structure will have the smaller γ in a certain threshold*.

### 6.4. The Influence of Interactive Rule Design for Resilience

**Figure 19:**
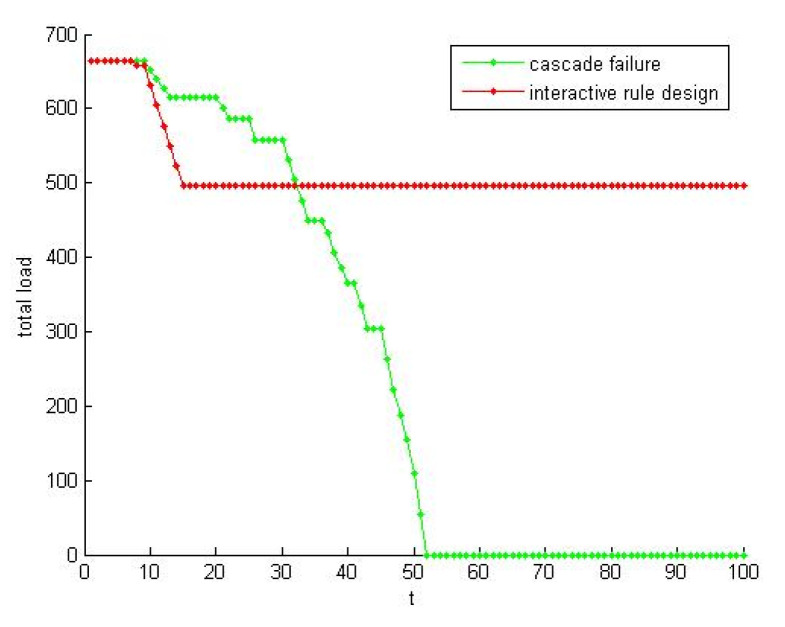
The effect of the interactive rule design.

In order to verify the influence of interactive rule design for resilience, the experiment of EO optimization in Section 5.2 was conducted for SN-1. At t = 0, the node *i* = 10 was selected as a failure. It has middle node degree which not only can avoid node degree being too large to lose randomness but also can make the network failure process be obvious. Setting the cycle number T = 100, the evolution curves of the total load in cascading failure were drawn. Contrast result before and after the optimization was shown in Figure 19. Using the EO, cascading failure extent decreased significantly. Take *t*_3_ = 52, R_P1_(SN-1) = 0.752, R_P2_(SN-1) = 0.873.

The EO algorithm is optimized for the nodes with the lowest fitness in the network. The setting of local fitness in the algorithm can make the optimization process of a single node give feedback to the whole network. This method makes improves enterprise satisfaction, and takes into account the saturation and the capability. This bottom-up optimization only changes individual variables. The variables of the network as a whole do not change.

SN-1 restores a new stable state through microconstant evolution of its self-organizing ability. Such emergence on the macrolevel is shown as supply chain resilience. This way can eliminate the influence brought by fluctuations, release energy, and then slow down the occurrence of large-scale collapse.

**Conclusion** **3.** *Interactive rule based on EO algorithm balance the relationship among the satisfaction, saturation and capacity. The method slows down the development of supercritical system. After adopting interaction rules, the resilience of SN increased by 16.09%*.

## 7. Conclusions

Our goal in this paper is to introduce the idea that an HRSN design based on SOC could improve resilience. We believe that this paper has achieved the following objectives.

First, this paper proposed a theory of the supply network resilience based on SOC.

The emergence of the supply network resilience is shown through self-organization evolution. In order to improve the efficiency and satisfaction, a slow self-organization evolution will enable supply developing from a critical state to a subcritical state. Cascading failure will ultimately occur because of the greater vulnerability. The supply fluctuates the critical state repeating the process after return to the state of low efficiency. In this state the supply network has the highest efficiency to balance the benefits and risks. We consider that resilient range is a self-organizing critical state.

Second, this paper proposed an idea of the design for supply network resilience based on SOC.

In the critical state, the efficiency is low. In supercritical, vulnerability is high. The goal of our design is to keep the SN within the critical state. It makes the vulnerability smallest, meanwhile the efficiency largest.

Third, this paper proposed specific methods of the design for supply network resilience.

At a macro level, network structure design makes the supply network has higher heterogeneity with smaller *γ* in a certain threshold. It can make the SN have better absorptive capacity to slow down the development to supercritical. The probability of the large-scale failure will decrease. At a micro level, the interactive rule design adopts the extremal optimization without adjusting parameters. It can make the supply network have better adaptive capacity to stay at the critical state.

Fourth, this paper proposed macro statistical indicator and micro performance indicator to measure the resilience.

In this paper, we study the supply network design for resilience mainly from the aspects of reducing the vulnerability due to cascading failure. This is achieved through a bottom-up design. Recovery measures require external intervention, and we have done research recently [35,36]. These studies are focused on how to mitigate epidemic impact on the healthcare supply network to control the outbreak of an epidemic.

The disasters caused by an epidemic are different from other disasters. There are two specific features: Long-term disruption and increasing propagation. The healthcare supply network is very vulnerable to collapse in this COVID-19 outbreak. Better supply chain management can enable health systems to reduce their supply expenses and cut forecasting errors optimizing supply chain management are significant. This research not only addresses the challenges brought by the pandemic, but also can be applied to other healthcare sectors, such as emergency medical services, blood supply, etc.

## Figures and Tables

**Table 1:** Parameters for supply network.

Denotation	Definition
*G* (*V*, *E*)	Supply network
*V*	The set of tier, *V* = {T_I_ | *I* = 1,2, …, *N*}, *T_I_* ∩ *T_J_* = *Ø*
*T* *_I_*	Tier *T_I_*
*N*	The total number of tiers in supply network
*M_I_*	The number of enterprises in *T_I_*
*E*	The set of relations between supply and demand, *E* = {<*i*,*j*> | *i* ∈ *T_I_*, *j* ∈ *T_I_*_+1_}
*i*	Enterprise *i*
*h_i_*	Load (operational ability of enterprise)
*z_i_*	Threshold (ceiling of operational ability)
*e_ij_* (*t*)	The expecting purchase quantity, Σ*e_ij_*(*t*) = *h_i_*(*t*)
*s_ij_* (*t*)	The actual purchase quantity, Σ*s_ij_*(*t*) ≤ *h_j_*(*t*)
*r_ij_* (*t*)	Satisfaction
*E_i_* (*t*)	The expecting to purchase quantity of enterprise *i*, *E_i_*(*t*) = *h_i_*(*t*)
*S_i_* (*t*)	The actual purchase quantity of enterprise *i*, *S_i_*(*t*) = Σ*s_ij_*(*t*) ≤ *h_i_*(*t*)
*R_i_* (*t*)	Satisfaction of enterprise *i*
*A_i_*	Saturation of enterprise *i*, *A_i_* = *h_i_*/*z_i_*
*k_i_*	The number of enterprises associated with enterprise *i*

**Table 2:** The parameters of two healthcare resources supply networks.

Parameters	SN-1	SN-2
Numbers of nodes *N*	51	79
Layers	5	5
Average degree <*k*>	2.47	2.38
The power law exponent of degree of distribution *γ*	2.2849	2.4355
Clustering coefficient *C*	0.023	0.0049
Density *D*	0.0392	0.0253
The shortest path *d*	2.96	2.63
